# Crossmodal illusions in neurorehabilitation

**DOI:** 10.3389/fnbeh.2015.00212

**Published:** 2015-08-10

**Authors:** Nadia Bolognini, Cristina Russo, Giuseppe Vallar

**Affiliations:** ^1^Department of Psychology, University of Milano-BicoccaMilan, Italy; ^2^Laboratory of Neuropsychology, IRCSS Istituto Auxologico ItalianoMilan, Italy; ^3^NeuroMi – Milan Center for NeuroscienceMilan, Italy

**Keywords:** crossmodal illusions, neurorehabilitation, multisensory, body representation, pain, motor disorders, sensory disorders

## Abstract

In everyday life, many diverse bits of information, simultaneously derived from the different sensory channels, converge into discrete brain areas, and are ultimately synthetized into unified percepts. Such multisensory integration can dramatically alter the phenomenal experience of both environmental events and our own body. Crossmodal illusions are one intriguing product of multisensory integration. This review describes and discusses the main clinical applications of the most known crossmodal illusions in rehabilitation settings. We consider evidence highlighting the contribution of crossmodal illusions to restore, at least in part, defective mechanisms underlying a number of disorders of body representation related to pain, sensory, and motor impairments in neuropsychological and neurological diseases, and their use for improving neuroprosthetics. This line of research is enriching our understanding of the relationships between multisensory functions and the pathophysiological mechanisms at the basis of a number of brain disorders. The review illustrates the potential of crossmodal illusions for restoring disarranged spatial and body representations, and, in turn, different pathological symptoms.

## Introduction

In everyday life, we are surrounded by a plethora of different sensory signals that concurrently hit our senses. Although our first-hand perception seems dominated by a single modality, commonly vision (Wade and Swanston, 2001), different sensory signals are simultaneously processed and integrated. In this way, without any conscious effort, perceptual and cognitive functions, as well as the underlying brain activity, are shaped by interactions between senses. The brain is endorsed with specialized neural and cortical mechanisms for the synthesis of information derived from the different senses into coherent, unitary representations that guarantee adaptive behavioral responses (Stein and Meredith, 1993; Driver and Noesselt, 2008). The primary advantage of multisensory integration consists in the enhancement of the salience of sensory stimuli, which, in turn, facilitates behavioral responses to them, as, for instance: fastening spatial orienting, improving memory and language comprehension, optimizing sensorimotor control (Bolognini et al., 2005; Alais et al., 2010). Multisensory facilitatory effects typically arise through the processing of congruent sensory signals. However, our brain also tends to impose coherence to discordant sensory information. This process may give rise to crossmodal illusions. Indeed, the way we perceive signals from our own body, or from the external space, can be conceived as the output of brain processes, based on an informed interpretation of the stimuli. Crossmodal illusions occur when what we sense with one modality affects what we experience in another modality. Seen in this perspective, crossmodal illusions represent perceptual strategies for dealing with inter-sensory conflicts, ultimately aimed at giving coherence to the on-going perceptual experience. Remarkably, this function does not result from inferential, or other higher-level “cognitive” processes, such as deploying a decision strategy for responding to ambiguous or conflicting experiences; rather, it is based on automatic multisensory interactions in the brain, which occur largely outside the realm of conscious perception. Two basic mechanisms have been proposed as to how multisensory interactions can affect perception. First, feedback projections from higher-order association regions of the cortex provide a way for information regarding one modality (e.g., vision) to influence sensory processing at putatively unisensory cortical stages, committed to a different modality (e.g., touch); alternatively, feed-forward anatomical connections between primary sensory-specific areas may allow a fast exchange of sensory information at lower-level stages of cortical processing (Driver and Noesselt, 2008; Cappe et al., 2009).

In recent years, there has been an explosion of research on crossmodal illusions, which has attempted at unravelling how multisensory interactions shape human perception and cognition. Crossmodal illusions have been applied also in clinical populations. In neuropsychological research, for instance, crossmodal illusions have been used to look for evidence of disrupted or abnormal multisensory integration in patients with acquired focal brain lesions, using anatomo-clinical data to infer associations between the site of a brain lesion, the resultant multisensory disorder, and findings from neuroimaging experiments in healthy humans (for a review of this line of research see Bolognini et al., 2013a). The present review considers another intriguing field of research, concerned with the possibility of using crossmodal illusions as tools for rehabilitation. Here, we focus on the two most renowned crossmodal illusions applied with clinical purposes, the Mirror Box and the Rubber Hand illusions, illustrating their efficacious use for the treatment of pain (phantom limb pain, complex regional pain syndrome, spinal cord injury), and post-stroke motor, spatial, and bodily impairments (hemiparesis, visuo-spatial neglect, somatoparaphrenia, anosognosia for hemiplegia, alien hand syndrome); finally, their potential for optimizing neuroprosthetics is discussed.

## Therapeutic Applications of Crossmodal Illusions

### Mirror Box Illusion

The Mirror Box Illusion (MBI) is one of the most famous crossmodal illusions (Ramachandran and Altschuler, 2009; Lamont et al., 2011). The MBI is generated by a visual-motor conflict: when participants move one hand in front of a parasagittal mirror, while the other hand is kept behind the mirror, hidden from view, they may experience the illusion of symmetrical bimanual movements, as well as other illusory kinaesthetic and motor effects on the hidden limb (e.g., Altschuler, 2005; Snijders et al., 2007; Romano et al., 2013). Watching the mirror reflection of own movements also increases the excitability in the motor cortex, ipsilateral to the moving hand (Garry et al., 2005). Additionally, the mismatch between the movement performed by participants and the movement they observe increases neuronal activity in areas associated with self-awareness and spatial attention (i.e., precuneus, posterior cingulate and posterior parietal cortices; Fink et al., 1999; Dohle et al., 2004; Matthys et al., 2009; Michielsen et al., 2011a; Nojima et al., 2012).

The first clinical application of the MBI has been for alleviating Phantom Limb Pain (PLP) after amputation (Ramachandran et al., 1995). Melzack (1992) first proposed a central role of multisensory integration of bodily signals by the so-called “neuromatrix” in the construction of the body image and the generation of pain. When, through the mirror reflection, the intact arm is superimposed on the phantom limb, patients report the sensation that they can move and relax the cramped phantom limb, and experience pain relief. In this framework, relief from PLP presumably results from the correction of the incongruence between motor output (intention) and sensory (proprioceptive) feedback, brought about by the visual input of movements of the missing limbs. As a consequence, the “latent” cortical map of the missing limb may be re-activated (Moseley et al., 2008; Ramachandran and Altschuler, 2009; Moseley and Flor, 2012). Moreover, the sensory-motor conflict generated by the MBI can modulate motor cortical excitability, which is altered in many patients suffering from chronic pain of central origin (Lefaucheur, 2013; Bolognini et al., 2015). However, not always moving the phantom limb diminishes PLP: in some amputees the phantom movement can actually increase PLP (cramping sensations). Therefore, an alternative version of the MBI has been developed, whose main feature is that patients look at the mirror reflection of touches applied to the intact hand, while receiving touches on the stump positioned behind the mirror (Schmalzl et al., 2013). The induction of illusory touch on the phantom hand effectively reduces PLP in patients who do not respond to the standard MBI. Hence, different inter-sensory conflicts may be appropriate for amputees with different types of phantom sensations: while the MBI is effective for reinstalling voluntary movements of paralyzed phantoms, and release concomitant clenching sensations, the visual-tactile version of the MBI is useful for patients who can voluntarily move their phantom, but tend to experience a concomitant increase in cramping sensations (Schmalzl et al., 2013).

Besides PLP, successful use of MBI has been reported in patients with other pain syndromes, such as the Complex Regional Pain Syndrome, and even in sensory re-education of severe hyperesthesia after hand injuries (Moseley and Flor, 2012). The finding of analgesic effects of the MBI across different pain disorders is suggestive of a common pathophysiological mechanism, linked to defective or altered multisensory processing, which can be regulated by the sensory-motor conflict of the MBI. Chronic pain is indeed associated with the disruption of a range of multisensory body-related cortical representations, which, in turn, reflects maladaptive neuroplastic changes in the brain.

One recent development of MBI for pain relief concerns its combined use with transcranial Direct Current Stimulation (tDCS) of the motor cortex. Different physiological mechanisms mediate the analgesic action of motor cortex stimulation by tDCS, which share some similarities with those of the MBI, namely: modulation of motor cortex excitability and changes in perceptual and emotional processing of the pain experience (Brunoni et al., 2012; Knotkova et al., 2013). Therefore, the use of tDCS as an add-on intervention to MBI could potentiate its antalgic action. This strategy was applied in patients with neuropathic pain following Spinal Cord Injury (Soler et al., 2010): tDCS over the motor cortex was delivered during a modified version of the MBI, consisting in a virtual reality procedure to induce a visual illusion of walking. The combined use of tDCS and MBI reduced the overall severity of pain, as well as improved various subtypes of neuropathic pain (continuous and paroxysmal pain, mechanical allodynia and dysaesthesias) in patients with spinal cord injury, with greater and longer-lasting effects than those induced by each single intervention (tDCS or MBI).

Another well-known application of the MBI is for the rehabilitation of hemiparesis in stroke patients (e.g., Altschuler et al., 1999; Dohle et al., 2009; Ramachandran and Altschuler, 2009; Michielsen et al., 2011b; Thieme et al., 2013). Although the precise mechanisms whereby MBI contributes to post-stroke motor recovery are still unclear, neuroimaging data highlight the key role of the sensory-motor mismatch (Michielsen et al., 2011a). Watching the reflection of self-generated movements in the mirror increases attentional demands for the integration of vision and proprioception, induced by the mirror, and neural activity in multisensory areas associated with self-awareness and spatial attention. These effects may translate into an increased awareness of the affected limb, which may counteract learnt non-use (Michielsen et al., 2011a, b). This hypothesis is also supported by evidence showing beneficial effects of the MBI on unilateral visuo-spatial neglect (Dohle et al., 2009). In this case, watching self-induced movements in the left side of space, contralateral to the side of the hemispheric lesion (contralesional), may facilitate leftward visuo-spatial orienting, impaired in left spatial neglect, and awareness of events occurring in the left, neglected, side of space. Noteworthy, self-observation in a mirror is also useful for ameliorating somatoparaphrenia, a somatic delusion usually following right-hemisphere lesions, which typically manifests as a defective sense of ownership of the patient’s left, contralesional, body parts (Fotopoulou et al., 2011; Jenkinson et al., 2013). Finally, “off-line” self-observation in a video replay reinstates motor awareness in patients with anosognosia for hemiplegia, speeding up recovery (Fotopoulou et al., 2009).

Recently, the MBI has been used to improve disorders of motor control, such as the Alien Hand Syndrome (AHS). The AHS is a higher-order disorder of motor control featured by involuntary, yet purposeful, movements of the affected limb, typically the hand, which may follow infarction in the vascular territory of anterior cerebral artery, midline tumors, and neurodegenerative illnesses. The impaired voluntary motor control in the AHS has been proposed to be due to a pathological neurofunctional disconnection between motor intentions and sensory information. Following this line of reasoning, in one patient with right AHS due to an intracerebral hemorrhage in the left fronto-parietal cortex, the MBI was used for restoring the congruency between motor intentions and visual feedback, consequently improving the voluntary fine motor control of the alien hand (Romano et al., 2014).

### Rubber Hand Illusion

The Rubber Hand Illusion (RHI) is another crossmodal illusion widely investigated in clinical settings (Botvinick and Cohen, 1998), although its application in rehabilitation is still in an early stage (Christ and Reiner, 2014). The RHI is induced by brushing a person’s hand, hidden from view, while synchronously brushing a visible rubber hand. This results in a sense of ownership of the rubber hand, and in a projection of sensations from the brushed rubber hand to the person. The RHI is also associated with a measurable proprioceptive drift in the perceived location of the hand towards the rubber hand, and changes in reaching movements performed with the stimulated hand (Tsakiris and Haggard, 2005; Kammers et al., 2010). The RHI has been used to uncover mechanisms of body ownership, the plasticity of body representations, and their dependency on multisensory integration of touch, proprioception, and vision. Deficient body ownership is the hallmark of somatoparaphrenia, which therefore represents the optimal condition for assessing the chance of manipulating and, possibly, restoring a deranged multisensory representation of the body concerned with self-representation and body-part ownership (Vallar and Ronchi, 2009). In one study in two right-brain-damaged patients with a stable somatoparaphrenia, we first applied the classical RHI paradigm to verify whether multisensory mechanisms supporting the sense of body-part ownership were disrupted, notwithstanding the patients’ pathologic delusion for their left hand (Bolognini et al., 2014; see also Jenkinson et al., 2013). Both patients proved to be susceptible to the RHI to the same extend as healthy subjects: this suggests undamaged multisensory mechanisms for the synthesis of bodily signals concerned with body ownership. In the light of such evidence, we investigated whether the RHI could also induce a remission of the delusional beliefs concerning the left hand. This hypothesis was tested with a modified version of the RHI, which consisted in stroking both the patient’s visible (disowned) left hand, and the right hand, hidden from view. Notably, patients could not report the feeling of being touched during stroking of their left hand, due to a co-occurring hemianesthesia. Our manipulation induced an immediate self-re-attribution of the left hand, with one patient showing a long-lasting remission of the somatic delusion. This evidence indicates that the multisensory representation of the body concerned with ownership, deranged as a somatic delusion in somatoparaphrenia, is not completely lost, as indicated by its restoration by multisensory bodily stimulations in the form of the RHI, which allows regaining the sense of ownership of the disowned hand.

The use of RHI in the Complex Regional Pain Syndrome (Reinersmann et al., 2013) and in Cervical Spinal Cord Injury (Lenggenhager et al., 2013) is based on a similar line of reasoning. In both these conditions, the RHI can be used to improve tactile awareness and processing, likely by reactivating tactile memories, and restoring an altered bodily self-representation, that may follow impaired sensorimotor abilities. The RHI is also useful for the treatment of chronic pain in these disorders, considering the tight link between pain and body image distortions along with disruption of a range of body-related cortical representations (Lotze and Moseley, 2007).

The aim of using the RHI to strengthen spatial and body awareness has guided its application in patients with unilateral visuo-spatial neglect. In a right-brain-damaged patient, immediately after the induction of the RHI (with the rubber hand located in the left side of space), a short-lasting amelioration of left spatial neglect took place in letter cancellation and midline pointing tasks, with the patient experiencing a shift in the felt position of his right hand towards the left-sided rubber hand (Kitadono and Humphreys, 2007). The amelioration of visual neglect could be due to changes in the patient’s egocentric reference frames, brought about the RHI, by cueing spatial attention, by modifying visuo-motor mapping, or by a combination of these different mechanisms. The susceptibility of patients with spatial neglect to crossmodal illusions suggests that multisensory integration is largely spared in this neuropsychological syndrome (Bolognini et al., 2013a), despite the possible presence of modality-specific attentional disturbances, which may impact perceptual awareness in different sensory modalities (Vallar and Bolognini, 2014).

Another field of application of the RHI concerns neuroprosthetics, where the major goal is to develop artificial limbs that feel like a real part of the body. Here, the RHI was used to favor, in a simple and non-invasive way, the embodiment of the prosthesis, re-creating a coherent representation of the body, and producing tactile sensations in the prosthetic limb in amputees. Indeed, for a limb to be functionally useful one must be able to sense not only movements, but even touch; additionally, the acceptance of the prosthesis as one’s own body-part influences the overall well-being of the amputee (Gallagher and MacLachlan, 1999). In upper limb amputees, the RHI can be induced by simultaneously touching the stump and the finger of the prosthesis; this elicits an illusion of sensing touch on the artificial hand and a feeling of ownership of it (Ehrsson et al., 2008; Rosén et al., 2009). A similar approach was used in upper limb amputees with surgically redirected nerves (Marasco et al., 2011). Of note, such crossmodal manipulations with the prosthesis activate in amputees the same multisensory regions in the premotor cortex and the intraparietal sulcus involved, in healthy individuals, in the integration of visual, tactile and proprioceptive signals, on which the feeling of body ownership (Schmalzl et al., 2013), and phantom sensations (Bolognini et al., 2013b) are based. This supports the use of this crossmodal strategy to guide post-amputation cortical plasticity.

## Discussion

The examples considered in this review indicate that “tricking” the brain with simple crossmodal illusions may offer an effective strategy to boost and wake up its multisensory capabilities, which may be latent or weakened by a brain disease. In this way, inter-sensory conflicts can restore altered sensory and motor representations subtending various disorders of body awareness, body ownership, movement, sensibility, attention and pain. Overall, these findings suggest that the brain may preserve its natural and basic tendency to integrate information from different sensory modalities in many pathological conditions, with crossmodal illusions resulting from hardwired perceptual organizing strategies or principles that, in general, are adaptive and advantageous. Such strategies involve rules for modulating experiential responses to multisensory information and rules that deal with important regularities (O’Callaghan, 2008), as ways for coping with environmental and corporeal changes that may emerge in pathological conditions. Noteworthy, current evidence points to an overall spared ability of combining multisensory cues, and, in particular, inputs from a damaged sensory modality with inputs from a spared modality, at least in patients with focal brain lesions (Bolognini et al., 2013a). This is likely to be the case since multiple, parallel, cortical and subcortical pathways are available for multisensory integration to occur: accordingly, if a key node of a multisensory network is damaged, alternative spared pathways may be used to re-connect the sensory systems. This allows to re-arrange sensory interactions, in order to preserve an optimal, reliable, multisensory experience. Importantly, the effects of crossmodal illusions may be even stronger in patients with brain disease than in healthy subjects, likely because neurological patients may present with sensory or motor deficits (e.g., Burin et al., 2015) that could be more vulnerable to crossmodal interferences from the intact senses. Moreover, some evidence suggests that increasing the degree of inter-sensory conflict can even enhance brain responses to crossmodal illusions (Senna et al., 2015). Many other crossmodal illusions were tested in clinical populations, though not in rehabilitation settings, including: the Ventriloquism illusion in hemianopia and neglect (Bertelson et al., 2000; Leo et al., 2008), the Aristotle’s illusion in focal hand dystonia (Tinazzi et al., 2013), and the Sound-induced flash illusion in migraine (Brighina et al., 2015). They could offer novel frameworks for therapeutic developments.

## Conflict of Interest Statement

The authors declare that the research was conducted in the absence of any commercial or financial relationships that could be construed as a potential conflict of interest.
