# Corrigendum: Crossmodal illusions in neurorehabilitation

**DOI:** 10.3389/fnbeh.2016.00033

**Published:** 2016-03-01

**Authors:** Nadia Bolognini, Cristina Russo, Giuseppe Vallar

**Affiliations:** ^1^Department of Psychology, and NeuroMi – Milan Center for Neuroscience, University of Milano-BicoccaMilan, Italy; ^2^Laboratory of Neuropsychology, IRCCS Istituto Auxologico ItalianoMilan, Italy

**Keywords:** crossmodal illusions, neurorehabilitation, multisensory, body representation, pain, motor disorders, sensory disorders

This Corrigendum is issued because the Department of Psychology, and the NeuroMi – Milan Center for Neuroscience are part of, and belong to, the same institution, namely the University of Milano-Bicocca, Milan, Italy. They are not two different institutions, as we erroneously stated in the original manuscript: that erroneous statement may affect the funding of our institutions. Accordingly, Dr. Bolognini and Dr. Vallar have 2 (not 3) affiliations: University of Milano-Bicocca, and IRCCS Istituto Auxologico Italiano, Milan, Italy. We state that the correction and the revised information do not affect the scientific validity of the results.

## Author contributions

NB, CR, and GV have contributed equally to all stages of this work.

### Conflict of interest statement

The authors declare that the research was conducted in the absence of any commercial or financial relationships that could be construed as a potential conflict of interest.

